# Supplementary material for: Characterization of patients requiring inpatient hospital ethics consults- A single center study
Source: PLoS One. 2024 Apr 2;19(4):e0296763. doi: 10.1371/journal.pone.0296763 (PMC10986956; doi:10.1371/journal.pone.0296763)

Supplemental Figure 1: Number of ethics meetings for patients in each admission diagnosis group.


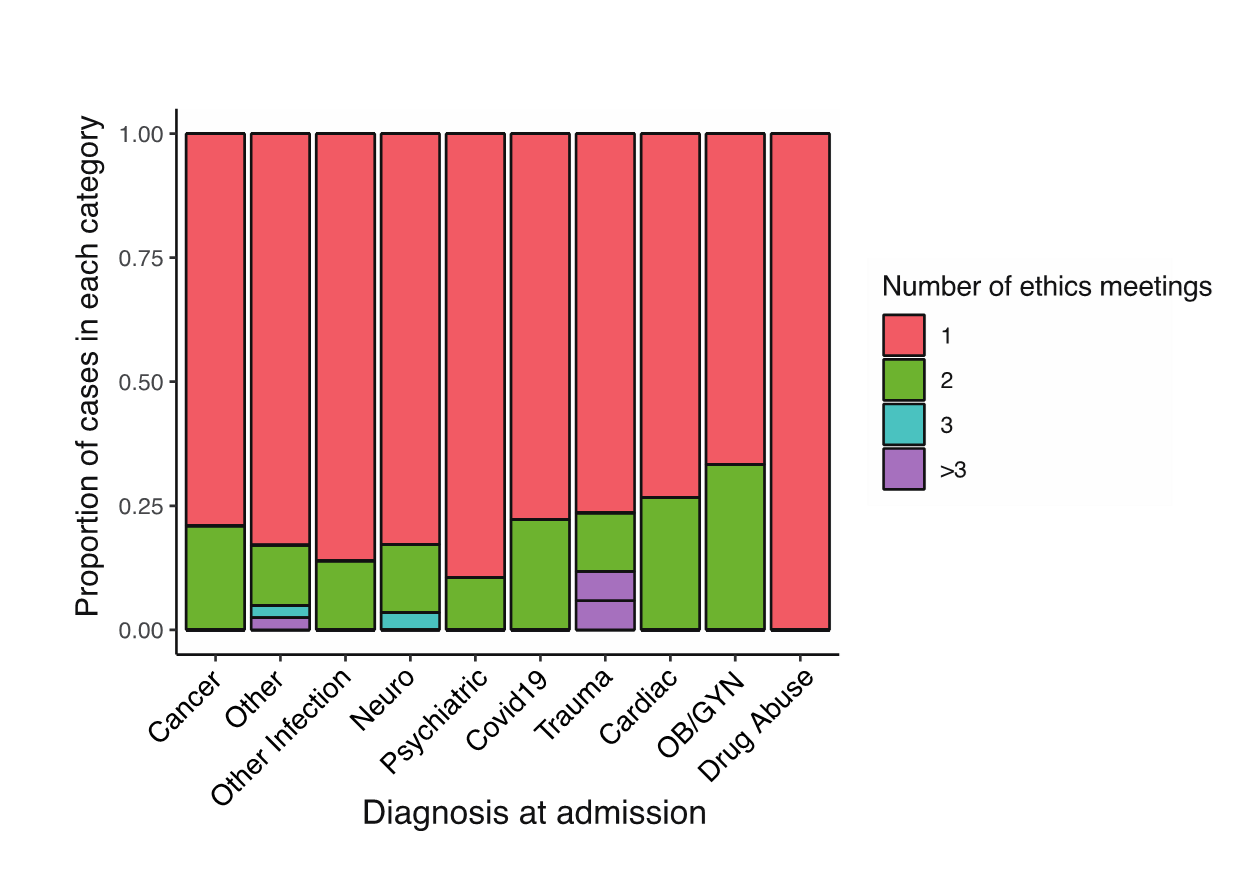

Supplement: S1 Fig — (DOCX) [file pone.0296763.s003.docx]
