# Supplementary material for: Characterization of patients requiring inpatient hospital ethics consults- A single center study
Source: PLoS One. 2024 Apr 2;19(4):e0296763. doi: 10.1371/journal.pone.0296763 (PMC10986956; doi:10.1371/journal.pone.0296763)
Supplement: S1 Table — P values indicate significant differences in demographic data between groups. (DOCX) [file pone.0296763.s004.docx]

Supplemental Table 1: Demographic and clinical data in different language groups. P values indicate significant differences in demographic data between groups.

|  | **English N (%)** | **Spanish N (%)** | **Vietnamese N (%)** | **English vs Spanish p-value** | **English vs Vietnamese p-value** | **English vs Spanish smd** | **English vs Vietnamese smd** |
| --- | --- | --- | --- | --- | --- | --- | --- |
| **Median Age (range)** | 61 (13-118) | 59 (23 - 118) | 75.5 (43 - 95) | 1 | 0.00017 | -0.12819 | -0.93441 |
| **Sex** |  |  |  |  |  |  |  |
| Female | 57 (34.3%) | 8 (26.7%) | 10 (41.7%) | 1 | 1 | 0.150272 | 0.082809 |
| Male | 108 (65.1%) | 22 (73.3%) | 15 (62.5%) |  |  |  |  |
| **Primary Admission Diagnosis Classifications** |  |  |  |  |  |  |  |
| Cancer | 33 (19.9%) | 5 (16.7%) | 4 (16.7%) | 0.4839 | 1 | 0.715732 | 0.791986 |
| Covid19 | 7 (4.2%) | 7 (23.3%) | 2 (8.3%) |  |  |  |  |
| Other Infection | 24 (14.5%) | 2 (6.7%) | 6 (25%) |  |  |  |  |
| Drug Abuse | 1 (0.6%) | 0 (0%) | 0 (0%) |  |  |  |  |
| Psychiatric | 14 (8.4%) | 1 (3.3%) | 1 (4.2%) |  |  |  |  |
| OB/GYN | 7 (4.2%) | 2 (6.7%) | 0 (0%) |  |  |  |  |
| Neuro | 23 (13.9%) | 3 (10%) | 2 (8.3%) |  |  |  |  |
| Cardiac | 12 (7.2%) | 2 (6.7%) | 0 (0%) |  |  |  |  |
| Trauma | 16 (9.6%) | 1 (3.3%) | 1 (4.2%) |  |  |  |  |
| Other | 27 (16.3%) | 5 (16.7%) | 8 (33.3%) |  |  |  |  |
| **Year** |  |  |  |  |  |  |  |
| 2017 | 1 (0.6%) | 0 (0%) | 2 (8.3%) | 1 | 0.4415 | 0.42707 | 0.515901 |
| 2018 | 49 (29.5%) | 5 (16.7%) | 4 (16.7%) |  |  |  |  |
| 2019 | 56 (33.7%) | 9 (30%) | 9 (37.5%) |  |  |  |  |
| 2020 | 30 (18.1%) | 10 (33.3%) | 6 (25%) |  |  |  |  |
| 2021 | 30 (18.1%) | 6 (20%) | 3 (12.5%) |  |  |  |  |
| **Religion** |  |  |  |  |  |  |  |
| Christian | 72 (43.4%) | 19 (63.3%) | 7 (29.2%) | 1 | 3.2E-07 | 0.511019 | 0.946968 |
| Buddhist | 1 (0.6%) | 0 (0%) | 7 (29.2%) |  |  |  |  |
| Muslim | 2 (1.2%) | 0 (0%) | 0 (0%) |  |  |  |  |
| Jewish | 1 (0.6%) | 0 (0%) | 0 (0%) |  |  |  |  |
| Other | 7 (4.2%) | 2 (6.7%) | 2 (8.3%) |  |  |  |  |
| unknown | 84 (50.6%) | 9 (30%) | 8 (33.3%) |  |  |  |  |
| **Capacity** |  |  |  |  |  |  |  |
| Yes | 22 (13.3%) | 2 (6.7%) | 3 (12.5%) | 1 | 1 | 0.231651 | 0.025784 |
| No | 138 (83.1%) | 27 (90%) | 20 (83.3%) |  |  |  |  |

Abbreviations: OB/GYN - Obstetrics and Gynecology; ICU – Intensive Care Unit; SMD - Standardized Mean Difference
