# Supplementary material for: Characterization of patients requiring inpatient hospital ethics consults- A single center study
Source: PLoS One. 2024 Apr 2;19(4):e0296763. doi: 10.1371/journal.pone.0296763 (PMC10986956; doi:10.1371/journal.pone.0296763)
Supplement: S2 Table — Sum of percentages may be greater than 100% for each diagnosis since patients receive multiple recommendations. (DOCX) [file pone.0296763.s005.docx]

Supplemental Table 2: Percentage of ethics recommendation outcomes in each diagnostic group. Sum of percentages may be greater than 100% for each diagnosis since patients receive multiple recommendations.

| **Principal Diagnosis** | **Make DNR** | **No escalation of care** | **Other** | **Transfer/Letter** | **Pursue Comfort Care** |
| --- | --- | --- | --- | --- | --- |
| Cancer | 30.23 | 30.23 | 46.51 | 9.3 | 37.21 |
| Cardiac | 50 | 25 | 43.75 | 18.75 | 37.5 |
| Covid19 | 55.56 | 33.33 | 38.89 | 16.67 | 38.89 |
| Drug Abuse | 0 | 0 | 100 | 0 | 0 |
| Neuro | 44.83 | 41.38 | 37.93 | 6.9 | 37.93 |
| OB/GYN | 0 | 11.11 | 77.78 | 11.11 | 0 |
| Other | 30.95 | 14.29 | 73.81 | 9.52 | 21.43 |
| Other Infection | 27.78 | 30.56 | 63.89 | 16.67 | 30.56 |
| Psychiatric | 26.32 | 10.53 | 78.95 | 15.79 | 10.53 |
| Trauma | 22.22 | 27.78 | 61.11 | 5.56 | 44.44 |
