# Supplementary material for: Characterization of patients requiring inpatient hospital ethics consults- A single center study
Source: PLoS One. 2024 Apr 2;19(4):e0296763. doi: 10.1371/journal.pone.0296763 (PMC10986956; doi:10.1371/journal.pone.0296763)
Supplement: Attachment — Submitted filename: Mahadevan_et_al_reviewer_responses_2.docx [file pone.0296763.s007.docx]

**2^nd^ round Responses to reviewer comments for “Characterization of Patients Requiring Inpatient Hospital Ethics Consults- A Single Center Study”**

**1. Kindly reformat your Abstract to a 'structured abstract' with sections: "Introduction or Background, Methods, Results, Conclusions.**

We thank the editor for this comment and have structured our abstract to include the requested sections.

**2. In the Methods Sections of the Body of the manuscript, "the variables to be included in the Cox proportional hazards model should not be predetermined or pre-mentioned, or you 'could could state that you used covariate with statistical significance by univariate analysis in the methods."**

Thank you for this suggestion. We did not pre-select variables to include in our multivariate cox proportional hazards model. As previously stated in the rebuttal and summarized by the reviewer, we included features in the multivariate model based on the significance results of univariate testing. We have modified the language in our methods section to reflect that we performed covariate testing using the statistical significance of univariate analyses for selecting features.

**3. Also, "use of covariate such as the languages or the age should only be included in the results."**

We thank the reviewer for this suggestion and have modified the text to only mention the use of language and age as covariates in the results section of the manuscript.
